# Maternal Psychological Distress and Parent-Reported Child Behavioural and Emotional Outcomes Among Mothers of Children with Autism Spectrum Disorder: A Cross-Sectional Study

**DOI:** 10.3390/healthcare14172816

**Published:** 2026-09-02

**Authors:** Khulud Ahmad Rezq, Haia Mahdi Hindi Albalawi, Hanan F. Alharbi

**Affiliations:** 1 Department of Community and Psychiatric Mental Health Nursing, Faculty of Nursing, University of Tabuk, Tabuk 47512, Saudi Arabia; 2 Department of Maternal and Child Health of Nursing, Faculty of Nursing, University of Tabuk, Tabuk 47512, Saudi Arabia; h.malbalawi@ut.edu.sa; 3 Maternity and Pediatric Nursing Department, Faculty of Nursing, Princess Nourah bint Abdulrahman University, Riyadh 11564, Saudi Arabia; hfalharbi@pnu.edu.sa

**Keywords:** autism spectrum disorder, anxiety, depression, stress, psychological, child behavior

## Abstract

**Background**: Mothers of children with autism spectrum disorder (ASD) face numerous challenges in meeting their children’s needs. This study aimed to assess parent-reported child behavioural and emotional outcomes and the levels of anxiety, depression, and stress among mothers of children with ASD. It also explored the correlations between parent-reported child behavioural and emotional outcomes and maternal mental health, as well as potential factors associated with these outcomes. **Methods**: A cross-sectional study was conducted between 30 December 2022 and 23 March 2023. An online questionnaire was distributed through autism associations across Saudi Arabia. The survey included (1) demographic data; (2) the Autism Family Experience Questionnaire (AFEQ) for children’s behavioural and emotional outcomes; and (3) the Depression, Anxiety, and Stress Scale-21 (DASS-21). Statistical analyses were performed using Jamovi software. **Results**: The study included 218 mothers of children with ASD. Extremely severe anxiety was reported by 41.3% of mothers, while 30.7% had normal stress and 30.7% had normal depression scores. The mean AFEQ symptom score was 37.4 ± 5.02. Univariate analysis revealed significant associations between parent-reported child behavioural and emotional outcomes and maternal mental health. In the multivariate analysis, higher family income (5000–10,000 SAR, *p* < 0.001; >10,000 SAR, *p* = 0.009) was associated with more favourable outcomes, while having more than one child with ASD was associated with less favourable outcomes (*p* = 0.015). Anxiety was positively associated with having two (*p* = 0.003) or three children (*p* = 0.029), while depression was negatively associated with having more than three children (*p* = 0.001). **Conclusions**: Higher family income was associated with more favourable parent-reported child behavioural and emotional outcomes, whereas having more than one child with ASD was associated with less favourable outcomes and higher maternal anxiety.

## 1. Introduction

Autism spectrum disorder (ASD) is a neurodevelopmental disorder associated with persistent difficulties in social communication and social interaction, together with restricted, repetitive patterns of behaviour, interests, or activities. The clinical presentation varies considerably among individuals, with symptoms ranging from mild to severe, and the disorder typically begins during early childhood and may persist into adulthood [1,2,3]. Globally, the prevalence of ASD has increased, with an estimated prevalence of approximately one in every 160 children, particularly among males [4]. Similarly, the reported prevalence in Arab countries ranges from 1.4 to 29 per 10,000 individuals [5], while the estimated prevalence in Saudi Arabia is 2.8 per 1000 individuals [6].

Many studies have found that parents of children with ASD experience higher levels of stress, depression, and anxiety than parents of typically developing children [7,8,9]. Compared with parents of children with other chronic conditions, such as diabetes mellitus and Trisomy 21, parents of children with ASD, particularly mothers, report significantly greater psychological distress and higher levels of parenting stress [10,11]. Moreover, parents of children with ASD are at increased risk of developing mental health disorders, including recurrent depressive disorder and anxiety disorders [12]. They also tend to report poorer quality of life than both the general population and mothers of children with intellectual disabilities [8,9].

These adverse psychological outcomes may be explained by the substantial caregiving demands associated with raising a child with ASD. Parents are often required to prioritise their child’s daily care, therapeutic interventions, and educational needs over other family responsibilities [13]. In addition, children with ASD generally require more caregiving time than typically developing children, including throughout the school years [14,15]. Behavioural and communication challenges associated with ASD may further affect the ability of children to adapt to their home, school, and social environments [16]. Collectively, these challenges increase the economic burden on families and reduce caregivers’ quality of life [17,18,19].

Mothers of children with ASD in Saudi Arabia may face additional challenges due to the country’s cultural, social, and healthcare contexts. Religious and cultural beliefs may influence how families perceive and cope with an ASD diagnosis, whereas social stigma may limit access to social assistance and community engagement [20,21]. Furthermore, access to specialised autism diagnostic and intervention services varies by area in Saudi Arabia, and some families continue to face challenges due to service availability, awareness, and financial constraints [22,23]. These contextual variables may affect maternal psychological well-being and parents’ perceptions of their children’s behavioural and emotional outcomes.

However, evidence regarding the association between maternal psychological distress and parent-reported child behavioural and emotional outcomes remains limited in Saudi Arabia. Therefore, this study aimed to assess parent-reported behavioural and emotional outcomes among children with ASD and the levels of anxiety, depression, and stress among their mothers. The secondary objectives were to examine the association between parent-reported child behavioural and emotional outcomes and maternal anxiety, depression, and stress, as well as to identify demographic and socioeconomic factors associated with both maternal psychological distress and parent-reported child behavioural and emotional outcomes.

## 2. Materials and Methods

### 2.1. Research Design and Setting

This cross-sectional study was conducted between 30 December 2022 and 23 March 2023. Data were collected using an online questionnaire distributed through autism associations across different regions of Saudi Arabia.

### 2.2. Respondent and Sampling

A purposive sampling technique was employed to collect data by reaching out to autism associations across all regions of Saudi Arabia. The study included mothers aged 18 years or older who had a child diagnosed with autism spectrum disorder (ASD). Mothers with a self-reported diagnosis of a mental illness, regardless of treatment status, were excluded to minimise potential confounding from pre-existing psychiatric disorders. Mothers without a previous diagnosis, including those with undiagnosed psychological symptoms, remained eligible for participation. Participants were recruited through official autism associations that maintain registries of families receiving autism-related services. These associations distributed the questionnaire through email lists and social media, covering all Saudi regions to ensure national representation. The minimum sample size was calculated using G-Power 3.1 software [19], which indicated a minimum required sample size of 180 participants. This calculation assumed a medium effect size (f^2^ = 0.15), 80% power, and a significance level of 0.05. To account for potential participants who might not meet the inclusion criteria, the sample size was increased by 20%, resulting in a total target of 216 participants.

### 2.3. Instrument and Data Collection

Data were collected using an online questionnaire consisting of three sections: (1) general demographic and baseline characteristics of the participants, including age, occupation, educational level, nationality, marital status, monthly family income, number of children per family, and number of children with ASD in the family, with monthly family income categorised as low (<5000 SAR), moderate (5000–10,000 SAR), or high (>10,000 SAR), based on national benchmarks and a 2018 survey reporting a median household income of 11,984 SAR [20]; (2) questions related to the Children’s Symptoms Scale of the Autism Family Experience Questionnaire (AFEQ); and (3) the Depression, Anxiety, and Stress Scale-21 (DASS-21). The Children’s Symptoms Scale of the AFEQ is a validated instrument consisting of 12 items developed by Leadbitter et al. in 2018 [20,21] to evaluate parents’ perceptions of the emotions and behaviours of their children with ASD. As no validated Arabic version of the scale was available at the time of this study, the instrument was translated into Arabic and culturally adapted by the research team before data collection. The translated version was subsequently reviewed by an expert in child health nursing and an expert in the English language to ensure linguistic accuracy, conceptual equivalence, and cultural appropriateness for use among Arabic-speaking mothers of children with ASD. The scale was rated on a five-point Likert scale, ranging from one (always) to five (never). The total score ranged from 12 to 60 points, with higher scores indicating poorer outcomes, while lower scores reflected more favourable outcomes. The scale includes six positively worded items and six negatively worded items, with the latter reverse-scored. In the present study, the AFEQ Children’s Symptoms Scale demonstrated acceptable internal consistency (Cronbach’s α = 0.75).

DASS-21 scale: We used the Arabic version of the DASS-21 scale, a validated and reliable tool for assessing mental health, which has demonstrated strong psychometric properties in Arabic-speaking populations [22]. The scale includes 21 items that measure three subscales: anxiety, stress, and depression. Each item was rated on a four-point Likert scale ranging from 0 (never) to 3 (almost always). The anxiety score was calculated as the sum of items 2, 4, 7, 9, 15, 19, and 20. The stress score was calculated as the sum of items 1, 6, 8, 11, 12, 14, and 18. The depression score was calculated as the sum of items 3, 5, 10, 13, 16, 17, and 21. In the present study, the Arabic version of the DASS-21 demonstrated excellent internal consistency for the total scale (Cronbach’s α = 0.94), with Cronbach’s α values of 0.87, 0.84, and 0.87 for the anxiety, stress, and depression subscales, respectively.

### 2.4. Statistical Analysis

Data were analysed using Jamovi software (version 2.4.8.0). Categorical variables are presented as frequencies and percentages, whereas continuous variables are summarised as means with standard deviations or medians with interquartile ranges, as appropriate. The normality of the data was assessed using the Shapiro–Wilk test, which indicated that the data were not normally distributed. Spearman’s rank correlation coefficient was used to examine correlations between variables. Univariate linear regression analyses were initially performed to examine the association between each independent variable and the study outcomes. Variables with statistically significant associations in the univariate analyses were subsequently entered into multivariate linear regression models to identify independent factors associated with the outcomes while controlling for potential confounding factors. Separate regression models were constructed for the AFEQ Children’s Symptoms Scale score and for the maternal depression, anxiety, and stress scores. In the model with the AFEQ Children’s Symptoms Scale score as the dependent variable, maternal depression, anxiety, and stress scores were included as independent variables to examine whether maternal psychological distress was associated with parent-reported child behavioural and emotional outcomes. The results were considered statistically significant when *p*-values were less than 0.05.

## 3. Results

**Figure 1:**
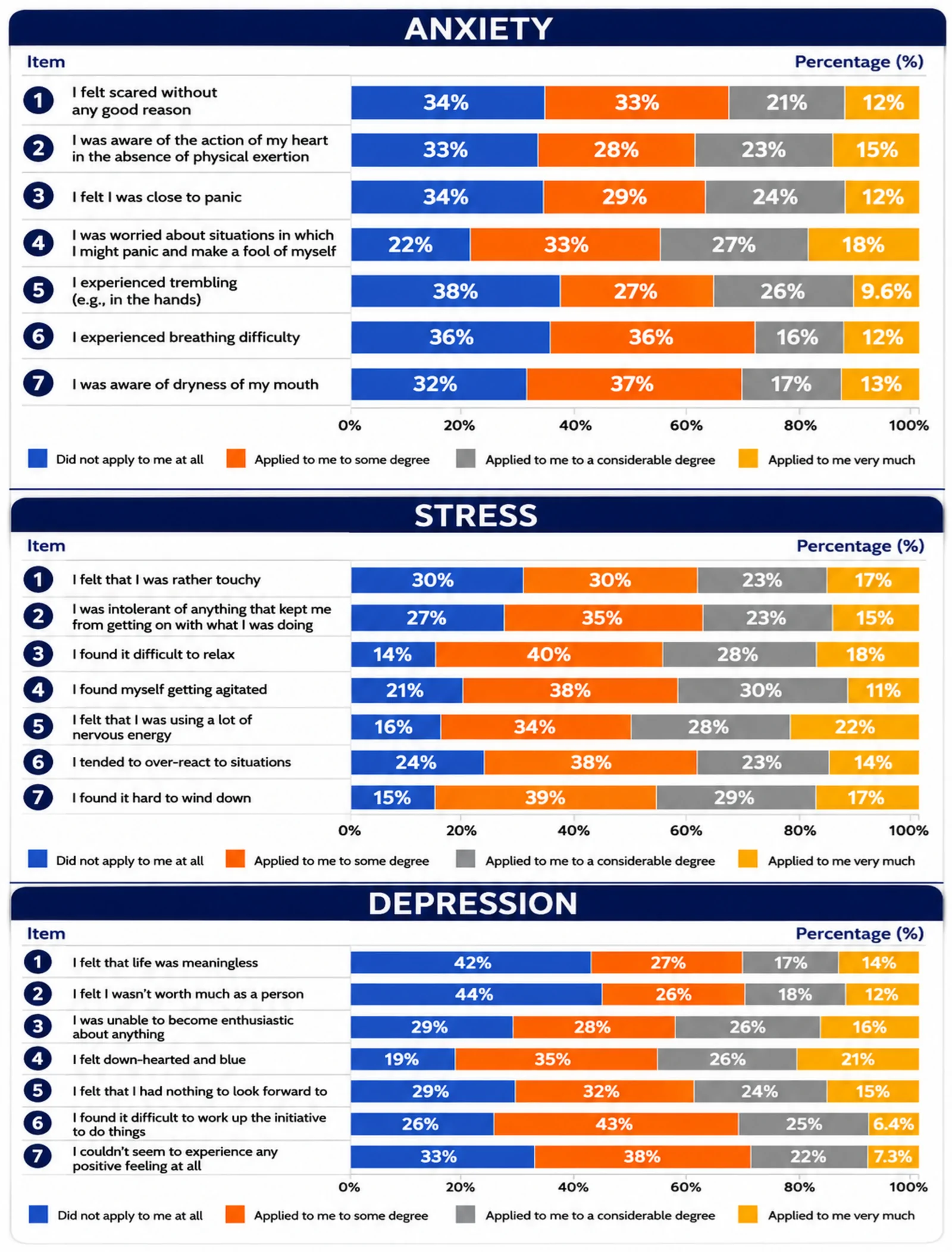
DASS-21 response distribution across items and categories.

**Figure 2:**
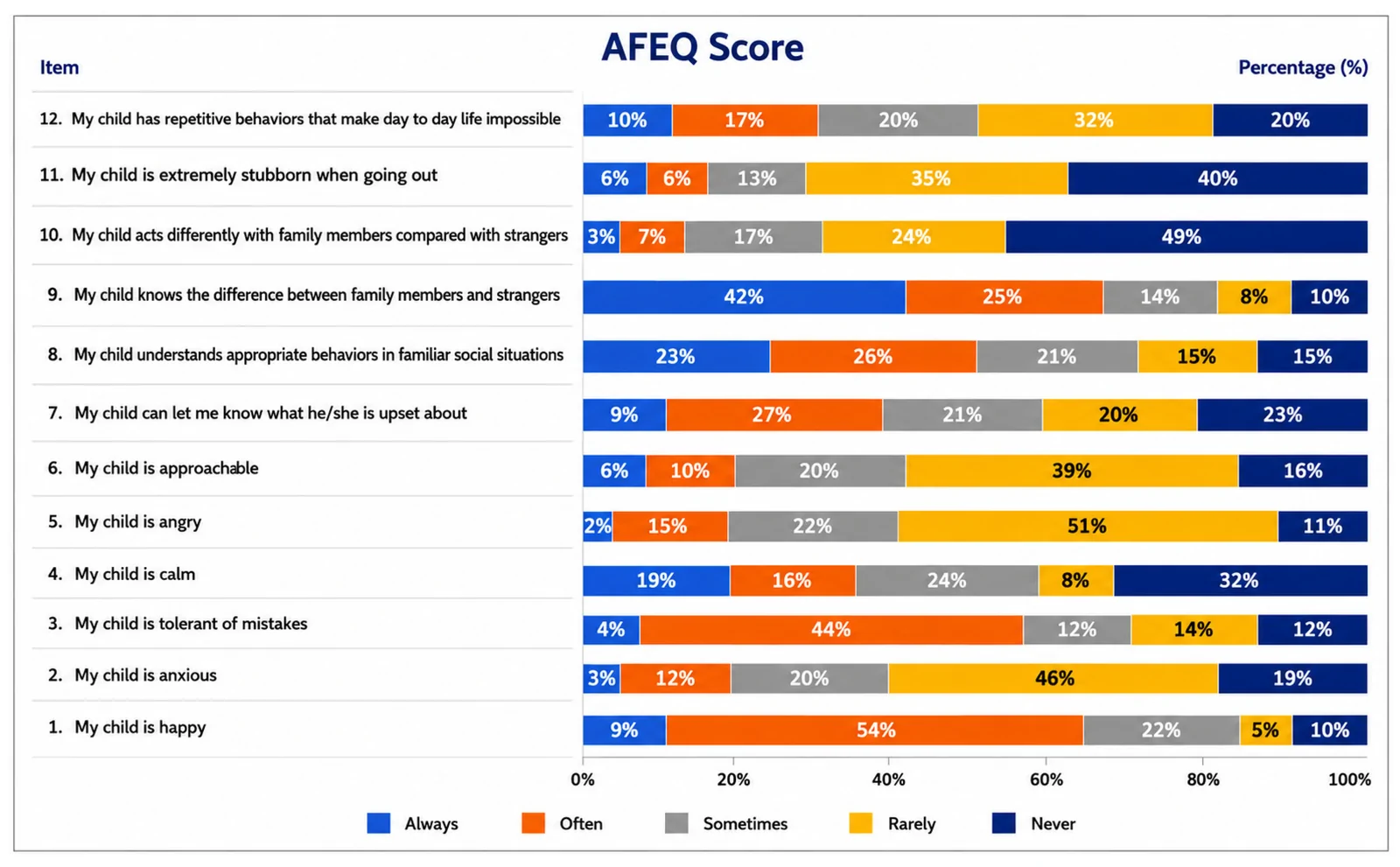
Distribution of AFEQ scores: parental perceptions of autism-related behaviours.

Our study included 218 mothers of children with ASD. Most of them were 30–39 years of age (40.4%), were Saudi nationals (92.2%), had a bachelor’s degree (52.8%), were married (89.9%), and were unemployed (57.3%). A total of 37.2% had more than three children, while 94.5% had only one child with ASD. Participants from the northern region represented 62.8% of the sample, which may reflect higher engagement from autism associations in that area. Additionally, 42.2% reported a monthly family income between 5000 and 10,000 Saudi Riyals. Table 1 shows the full details of the baseline characteristics of the included participants. About 41.3% of the total sample had extremely severe levels of anxiety, with a mean score of 16.2, while 30.7% had normal levels of both stress and depression, with mean scores of 19.4 and 16.4, respectively. The mean AFEQ score was 37.4 ± 5.02. Table 1 and Figure 1 and Figure 2 show the full details of the DASS-21 and AFEQ results.

**Figure 3:**
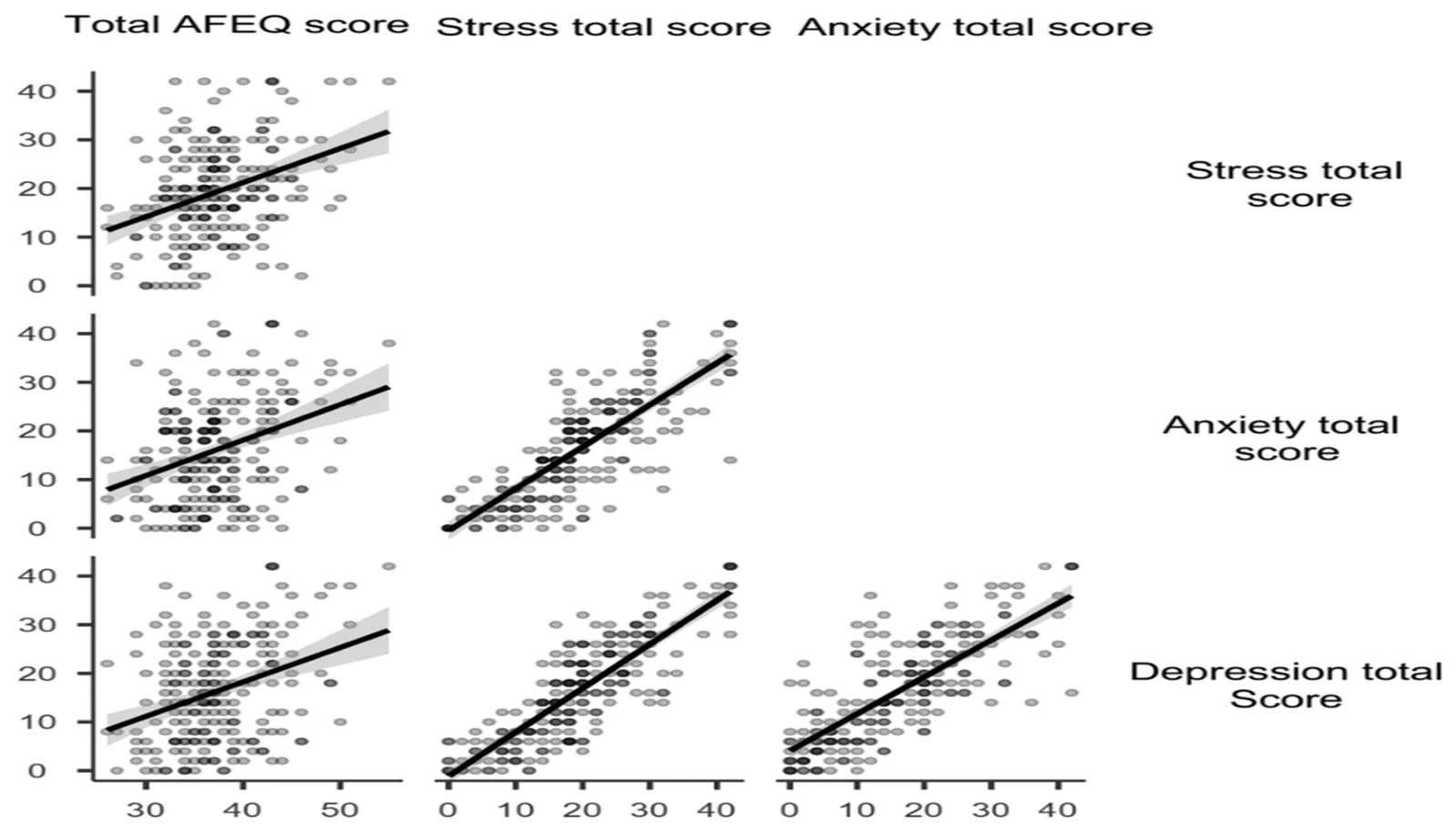
Correlation between AFEQ scores and DASS-21 subscale scores (stress, anxiety, and depression).

Our correlation analysis found a significant positive correlation between the AFEQ Children’s Symptoms Scale score and the levels of depression, anxiety, and stress. Family income showed significant positive correlations with maternal age (*p* = 0.018), educational level (*p* < 0.001), and number of children (*p* = 0.002), while it showed significant negative correlations with the AFEQ score (*p* < 0.001), stress score (*p* = 0.004), anxiety score (*p* < 0.001), and depression score (*p* = 0.002). Maternal age showed a significant negative correlation with educational level (*p* = 0.009) and a significant positive correlation with the number of children (*p* < 0.001). Finally, there was a significant negative correlation between educational level and the number of children (*p* = 0.032). Table 2 and Figure 3 show the full details.

In our univariate regression analysis of factors associated with the AFEQ Children’s Symptoms Scale score, we found significant negative associations with employment (*p* = 0.02), Saudi nationality (*p* = 0.01), monthly income between 5000 and 10,000 Saudi Riyals (*p* < 0.001), and monthly income greater than 10,000 Saudi Riyals (*p* < 0.001), while the AFEQ score showed significant positive associations with the presence of more than one child with ASD (*p* = 0.01), stress score (*p* < 0.001), anxiety score (*p* < 0.001), and depression score (*p* < 0.001). In the multivariate regression analysis, the AFEQ score had significant negative associations only with monthly income between 5000 and 10,000 Saudi Riyals (*p* < 0.001) and monthly income greater than 10,000 Saudi Riyals (*p* = 0.009), while it had a significant positive association only with the presence of more than one child with ASD (*p* = 0.015). Table 3 shows the full details.

For the stress score, the univariate regression analysis showed a negative association only with monthly family income greater than 10,000 Saudi Riyals (*p* = 0.001), while the stress score had positive associations with the AFEQ score (*p* < 0.001), depression score (*p* < 0.001), and anxiety score (*p* < 0.001). However, in the multivariate regression analysis, the stress score was significantly positively associated only with depression (*p* < 0.001) and anxiety scores (*p* < 0.001). Table 4 and Table 5 show the full details.

Regarding factors associated with anxiety, the univariate regression analysis revealed a significant negative association only with monthly family income greater than 10,000 Saudi Riyals (*p* < 0.001), while the anxiety score was positively associated with the presence of two children (*p* = 0.012), the AFEQ score (*p* < 0.001), the depression score (*p* < 0.001), and the stress score (*p* < 0.001). However, the multivariate regression analysis showed significant positive associations with the presence of two children (*p* = 0.003), the presence of three children (*p* = 0.029), the depression score (*p* < 0.001), and the stress score (*p* < 0.001). Table 6 and Table 7 show the full details.

Table 8 shows that most mothers were aged 30–39 years (40.4%), married (89.9%), unemployed (57.3%), Saudi nationals (92.2%), and held a bachelor’s degree (52.8%). Across depression severity levels, these sociodemographic characteristics remained relatively stable. However, differences were observed in the number of children and family income. Mothers with more than three children were more common in the normal and mild depression groups but became less common with increasing depression severity. In contrast, lower family income (<5000 SAR) showed an increasing pattern as depression severity increased, particularly in the extremely severe group. Overall, the findings suggest limited variation in most sociodemographic factors across depression levels, with noticeable trends only in the number of children and family income.

Univariate analysis identified several factors significantly associated with depression scores (Table 9). Higher income (5000–10,000 SAR and >10,000 SAR) was associated with lower depression scores, and having more than three children was also associated with lower scores. Postgraduate education reached significance at the conventional threshold (*p* = 0.049), though this was borderline and did not persist after adjustment. Both remaining psychological variables (stress score and anxiety score) were also significantly associated with depression scores. In the multivariate analysis, the results became more selective. After adjusting for all variables, only two factors remained significantly associated with depression scores: anxiety score (β = 0.671, *p* < 0.001) and having more than three children (β = −3.97, *p* = 0.001). Postgraduate education, income, and stress were no longer significant, indicating that their effects were attenuated after controlling for anxiety and other variables (Table 9).

## 4. Discussion

This study aimed to investigate the association between parent-reported child behavioural and emotional outcomes and maternal psychiatric manifestations, as well as to identify factors associated with these outcomes and maternal psychological distress. We found significant positive correlations between psychiatric manifestations and parent-reported child behavioural and emotional outcomes. In the multivariate regression analyses, higher family income was associated with more favourable parent-reported child behavioural and emotional outcomes, whereas having more than one child with ASD was associated with less favourable outcomes. Anxiety was positively associated with having two or three children, whereas having more than three children was negatively associated with depression scores. Positive associations between parent-reported child behavioural and emotional outcomes and psychiatric distress were observed only in the univariate regression analyses and were not retained in the multivariate regression analyses. Many studies have found that depression, anxiety, and stress are elevated among mothers of children with ASD [8,10,23]. Overall, these findings suggest that mothers of children with ASD are at an increased risk of psychological distress. Additionally, Alansari et al. found that mothers of children with ASD had poorer mental and physical health outcomes [9].

Regarding stress, in a Saudi Arabian study, Alghamdi et al. found that stress was the predominant psychological manifestation and was significantly more common among mothers of children with ASD than among mothers of typically developing children, whereas there was no significant difference between the two groups regarding depression or anxiety [24]. They explained this by suggesting that depression and anxiety occurred mainly during the early stages of the disorder, while stress persisted due to the continuous presence of stressful events [24,25]. Moreover, another Saudi study showed that both maternal and paternal stress were positively associated with ASD symptom severity in children [26]. Similarly, other studies also reported increased levels of stress among mothers of children with ASD [27,28].

Regarding anxiety and depression, a study conducted in China found that both anxiety and depression were significantly increased among mothers of children with ASD [29]. Similarly, another study performed in Saudi Arabia found that depression was increased among mothers of children with ASD compared with mothers of typically developing children [23]. In our study, anxiety was the most common psychiatric manifestation, with 41.3% of mothers reporting extremely severe anxiety, whereas 30.7% had normal stress and depression scores. However, our study did not include a comparison group of mothers of typically developing children. This finding is supported by Al-Adawi et al., who reported that anxiety was more prominent than other psychiatric manifestations in Arab and Islamic populations because of its somatic expression [30]. Although previous studies have reported equivocal findings regarding the predominant psychiatric manifestation, nearly all have concluded that mothers of children with ASD are at a higher risk of psychological distress, regardless of its specific manifestation [7,8,9,23,24,25]. The discrepancies between the findings of the present study and those reported from China and the United States may be attributed to differences in family structures, healthcare systems, cultural attitudes, and the availability of autism-related support services. In Saudi Arabia, mothers are often the primary caregivers of children with ASD and may have additional caregiving responsibilities within the family. Maternal psychological well-being and parent-reported perceptions of children’s behavioural and emotional outcomes may also be influenced by cultural expectations, social stigma, and differences in access to specialised services. These contextual factors should be considered when comparing findings across countries.

Regarding the association between parent-reported child behavioural and emotional outcomes and maternal psychiatric manifestations, a study performed in the United States found that parental stress levels and children’s behaviour were correlated with each other [28]. Other studies found that depressive symptoms increased with greater ASD symptom severity [31,32]. This could be explained by the substantial challenges experienced by parents raising a child with ASD [33]. Family adaptation to these circumstances and the challenges of raising a child with a disability may take considerable time [34]; however, other studies found that the behaviour of children with ASD was significantly associated with parental mental illness during middle childhood [35] but not during the preschool phase [36]. Another study, however, found that caregiver stress levels were significantly associated with ASD symptom severity during the preschool period [37]. These equivocal findings are consistent with our results, in which significant correlations and associations were observed only in the univariate regression analyses and not in the multivariate analysis. Therefore, more longitudinal studies with longer follow-up periods are needed to establish these associations, as our study was cross-sectional.

Almansour et al. found that the number of children was not associated with the degrees of depression or anxiety; however, the scores were significantly increased among caregivers who had three children with ASD compared with those who had only one [7]. This finding was not in line with our results, which showed no significant association between the number of children with ASD and psychiatric manifestations in the multivariate regression analysis but did show a significant association between the number of children and both anxiety (positive association) and depression (negative association). Therefore, longitudinal comparative studies are needed to clarify these equivocal findings. Koziarz et al. found that family income was not associated with ASD symptom severity or symptom frequency in the multivariate regression analysis [38]; however, another study found that the severity of the symptoms was negatively correlated with family income [39], which was supported by previous evidence showing that lower family income is associated with a higher risk of psychiatric illness among children [40]. Lower family income was associated with a reduced ability to provide appropriate management and meet the needs of children with ASD [39], which is consistent with our findings. Moreover, a recent Saudi study by Rezq et al. found that greater social support was significantly associated with a better quality of life among mothers of children with ASD, while lower family income and having more than one child with ASD were linked to poorer maternal outcomes [41]. These findings further support the substantial psychosocial burden experienced by mothers caring for children with ASD and highlight the importance of supportive interventions and socioeconomic resources in improving maternal well-being.

This study is among the few to have examined the association between parent-reported child behavioural and emotional outcomes and maternal psychiatric manifestations across multiple regions in Saudi Arabia. Its broad geographical coverage enhances the representativeness of the findings within the national context. However, as a cross-sectional design was employed, causal relationships cannot be established, and there is a risk of reporting bias due to self-reported data. This underscores the need for future longitudinal studies that incorporate key variables such as the child’s age and duration since diagnosis. Additionally, the absence of detailed child-related clinical characteristics, including intellectual and language functioning, co-occurring conditions, and treatment history, represents a limitation of the present study, as these factors may influence parent-reported behavioural and emotional outcomes. Furthermore, the use of purposive sampling targeting individuals registered with and receiving follow-up from autism associations may introduce selection bias and limit the generalisability of the results to families not affiliated with such associations. Moreover, although mothers with diagnosed mental illness were excluded to minimise confounding, it is important to consider that some mental health issues may have developed as a result of the chronic stress of raising a child with ASD. Future longitudinal studies should explore this dynamic to better understand potential causality. Finally, further multicentre studies are recommended to validate and generalise these findings across different settings and populations.

## 5. Conclusions

Among Saudi mothers of children with ASD, anxiety was the most common psychological manifestation, whereas approximately one-third of mothers had normal stress and depression scores. Significant positive correlations between maternal psychological distress and parent-reported child behavioural and emotional outcomes were observed in the univariate analyses but were not retained in the multivariate models. Higher family income was associated with more favourable parent-reported child behavioural and emotional outcomes, whereas having more than one child with ASD was associated with less favourable parent-reported child behavioural and emotional outcomes. These findings highlight the importance of providing culturally appropriate psychological support and family-centred interventions for Saudi mothers of children with ASD.

## Figures and Tables

**Table 1:** Baseline characteristics of the study participants (N = 218).

Variable	Counts	% of Total
Maternal age		
less than 20 years	5	2.3%
20–29 years	60	27.5%
30–39 years	88	40.4%
More than 40 years	65	29.8%
Nationality		
Saudi	201	92.2%
Non-Saudi	17	7.8%
Educational level		
Less than high school education	30	13.8%
High school certificate	59	27.1%
Bachelor’s degree	115	52.8%
Postgraduate degree	14	6.4%
Marital status		
Married	196	89.9%
Divorced or separated	21	9.6%
Widow	1	0.5%
Occupational status		
Employed	93	42.7%
Unemployed	125	57.3%
Number of children with ASD		
One	206	94.5%
More than one	12	5.5%
Number of children		
One	28	12.8%
Two	53	24.3%
Three	56	25.7%
More than three	81	37.2%
Residency region		
Central	27	12.4%
Western	33	15.1%
Southern	12	5.5%
Eastern	9	4.1%
Northern	137	62.8%
Family income		
Less than 5000 Saudi Riyal	46	21.1%
5000–10,000 Saudi Riyal	92	42.2%
More than 10,000 Saudi Riyal	80	36.7%
Stress degrees		
Normal	67	30.7%
Mild	45	20.6%
Moderate	49	22.5%
Severe	40	18.3%
Extremely Severe	17	7.8%
Anxiety degrees		
Normal	54	24.8%
Mild	9	4.1%
Moderate	46	21.1%
Severe	19	8.7%
Extremely Severe	90	41.3%
Depression degrees		
Normal	67	30.7%
Mild	21	9.6%
Moderate	57	26.1%
Severe	34	15.6%
Extremely Severe	39	17.9%
Stress total score		
Mean (SD)	19.4	9.8
Median (IQR)	18	(14, 26)
Anxiety total score		
Mean (SD)	16.2	10.65
Median (IQR)	15	(8, 24)
Depression total score		
Mean (SD)	16.4	10.52
Median (IQR)	16	(8, 24)
Total AFEQ score		
Mean (SD)	37.4	5.02
Median (IQR)	37	(34, 40.8)

AFEQ = The Autism Family Experience Questionnaire; SD = standard deviation; IQR = interquartile range.

**Table 2:** Spearman’s correlation analysis between the AFEQ Children’s Symptoms Scale scores and maternal depression, anxiety, and stress scores.

Variable		Maternal Age	Educational Level	Number of Children	Family Income	Total AFEQ Score	Stress Total Score	Anxiety Total
Maternal age Less than 20 years = 5 (2.3%) 20–29 years = 60 (27.5%) 30–39 years = 88 (40.4%) More than 40 years = 65 (29.8%)	Spearman’s rho	—	—	—	—	—	—	—
	*p*-value	—	—	—	—	—	—	—
Educational level Less than high school = 30 (13.8%) High school certificate = 59 (27.1%) Bachelor’s degree = 115 (52.8%) Postgraduate degree = 14 (6.4%)	Spearman’s rho	−0.176	—	—	—	—	—	—
	*p*-value	* 0.009	—	—	—	—	—	—
Number of children One = 28 (12.8%) Two = 53 (24.3%) Three = 56 (25.7%) More than three = 81 (37.2%)	Spearman’s rho	0.456	−0.145	—	—	—	—	—
	*p*-value	* <0.001	* 0.032	—	—	—	—	—
Family income Less than 5000 Saudi Riyal = 46 (21.1%) 5000–10,000 Saudi Riyal = 92 (42.2%) More than 10,000 = 80 (36.7%)	Spearman’s rho	0.16	0.239	0.21	—	—	—	—
	*p*-value	* 0.018	* <0.001	* 0.002	—	—	—	—
Total AFEQ score Mean (SD) = 37.4 (5.02)	Spearman’s rho	0.047	−0.09	−0.065	−0.27	—	—	—
	*p*-value	0.488	0.186	0.34	* <0.001	—	—	—
Stress total score Mean (SD) = 19.4 (9.8)	Spearman’s rho	−0.026	0.079	−0.082	−0.196	0.319	—	—
	*p*-value	0.7	0.243	0.227	* 0.004	* <0.001	—	—
Anxiety total Mean (SD) = 16.2 (10.65)	Spearman’s rho	−0.116	0.1	−0.146	−0.257	0.296	0.796	—
	*p*-value	0.087	0.142	* 0.031	* <0.001	* <0.001	* <0.001	—
Depression Total Mean (SD) = 16.4 (10.52)	Spearman’s rho	−0.128	0.114	−0.219	−0.205	0.285	0.832	0.77
	*p*-value	0.059	0.093	* 0.001	* 0.002	* <0.001	* <0.001	* <0.001

* Statistically significant at *p* < 0.05. AFEQ = The Autism Family Experience Questionnaire; SD = standard deviation.

**Table 3:** Univariate and multivariate regression analyses of factors associated with AFEQ Children’s Symptoms Scale scores.

Factor	Univariate			Multivariate		
	β	95% CI	*p*	β	95% CI	*p*
Maternal age, marital status, education, number of children, residency (all NS)						
No significant univariate associations—none entered in multivariate model						
Occupational status (ref: unemployed)						
Employed	−1.59	[−2.93, −0.25]	0.020 *	−1.078	[−2.33, 0.18]	0.092
Nationality (ref: non-Saudi)						
Saudi	−3.25	[−5.72, −0.79]	0.010 *	−1.011	[−3.41, 1.39]	0.407
Number of children with ASD (ref: one)						
More than one	3.86	[0.96, 6.76]	0.009 *	3.284	[0.65, 5.92]	0.015 *
Family income (ref: <5000 SAR)						
5000–10,000 SAR	−4.10	[−5.79, −2.41]	<0.001 *	−2.905	[−4.61, −1.20]	<0.001 *
>10,000 SAR	−4.16	[−5.89, −2.43]	<0.001 *	−2.417	[−4.23, −0.60]	0.009 *
Psychological scales						
Stress total score	0.184	[0.12, 0.25]	<0.001 *	0.104	[−0.02, 0.23]	0.107
Anxiety total score	0.162	[0.10, 0.22]	<0.001 *	0.055	[−0.04, 0.15]	0.269
Depression total score	0.161	[0.10, 0.22]	<0.001 *	0.008	[−0.10, 0.12]	0.888

* Statistically significant at *p* < 0.05; β = unstandardised coefficient; NS = not significant; CI = confidence interval.

**Table 4:** Distribution of participants according to stress severity levels.

Variable	Normal (N = 67)	Mild (N = 45)	Moderate (N = 49)	Severe (N = 40)	Extremely Severe (N = 17)	Total (N = 218)
Maternal age						
less than 20 years	1.0 (1.5%)	2.0 (4.4%)	2.0 (4.1%)	0.0 (0.0%)	0.0 (0.0%)	5.0 (2.3%)
20–29 years	16.0 (23.9%)	12.0 (26.7%)	15.0 (30.6%)	13.0 (32.5%)	4.0 (23.5%)	60.0 (27.5%)
30–39 years	32.0 (47.8%)	17.0 (37.8%)	17.0 (34.7%)	16.0 (40.0%)	6.0 (35.3%)	88.0 (40.4%)
More than 40 years	18.0 (26.9%)	14.0 (31.1%)	15.0 (30.6%)	11.0 (27.5%)	7.0 (41.2%)	65.0 (29.8%)
Marital status						
Married	61.0 (91.0%)	41.0 (91.1%)	43.0 (87.8%)	36.0 (90.0%)	15.0 (88.2%)	196.0 (89.9%)
Divorced or separated	6.0 (9.0%)	4.0 (8.9%)	6.0 (12.2%)	4.0 (10.0%)	1.0 (5.9%)	21.0 (9.6%)
Widow	0.0 (0.0%)	0.0 (0.0%)	0.0 (0.0%)	0.0 (0.0%)	1.0 (5.9%)	1.0 (0.5%)
Occupational status						
Employed	29.0 (43.3%)	21.0 (46.7%)	22.0 (44.9%)	16.0 (40.0%)	5.0 (29.4%)	93.0 (42.7%)
Unemployed	38.0 (56.7%)	24.0 (53.3%)	27.0 (55.1%)	24.0 (60.0%)	12.0 (70.6%)	125.0 (57.3%)
Nationality						
Saudi	63.0 (94.0%)	41.0 (91.1%)	44.0 (89.8%)	37.0 (92.5%)	16.0 (94.1%)	201.0 (92.2%)
Non-Saudi	4.0 (6.0%)	4.0 (8.9%)	5.0 (10.2%)	3.0 (7.5%)	1.0 (5.9%)	17.0 (7.8%)
Educational level						
Less than high school education	11.0 (16.4%)	5.0 (11.1%)	6.0 (12.2%)	6.0 (15.0%)	2.0 (11.8%)	30.0 (13.8%)
High school certificate	19.0 (28.4%)	15.0 (33.3%)	9.0 (18.4%)	11.0 (27.5%)	5.0 (29.4%)	59.0 (27.1%)
Bachelor’s degree	35.0 (52.2%)	21.0 (46.7%)	30.0 (61.2%)	20.0 (50.0%)	9.0 (52.9%)	115.0 (52.8%)
Postgraduate degree	2.0 (3.0%)	4.0 (8.9%)	4.0 (8.2%)	3.0 (7.5%)	1.0 (5.9%)	14.0 (6.4%)
Number of children						
One	10.0 (14.9%)	5.0 (11.1%)	6.0 (12.2%)	6.0 (15.0%)	1.0 (5.9%)	28.0 (12.8%)
Two	14.0 (20.9%)	11.0 (24.4%)	11.0 (22.4%)	9.0 (22.5%)	8.0 (47.1%)	53.0 (24.3%)
Three	15.0 (22.4%)	11.0 (24.4%)	14.0 (28.6%)	11.0 (27.5%)	5.0 (29.4%)	56.0 (25.7%)
More than three	28.0 (41.8%)	18.0 (40.0%)	18.0 (36.7%)	14.0 (35.0%)	3.0 (17.6%)	81.0 (37.2%)
Number of children with ASD						
One	65.0 (97.0%)	41.0 (91.1%)	46.0 (93.9%)	38.0 (95.0%)	16.0 (94.1%)	206.0 (94.5%)
More than one	2.0 (3.0%)	4.0 (8.9%)	3.0 (6.1%)	2.0 (5.0%)	1.0 (5.9%)	12.0 (5.5%)
Family income						
Less than 5000 Saudi Riyal	11.0 (16.4%)	6.0 (13.3%)	10.0 (20.4%)	12.0 (30.0%)	7.0 (41.2%)	46.0 (21.1%)
5000–10,000 Saudi Riyal	25.0 (37.3%)	20.0 (44.4%)	26.0 (53.1%)	15.0 (37.5%)	6.0 (35.3%)	92.0 (42.2%)
More than 10,000 Saudi Riyal	31.0 (46.3%)	19.0 (42.2%)	13.0 (26.5%)	13.0 (32.5%)	4.0 (23.5%)	80.0 (36.7%)
Residency region						
Central	6.0 (9.0%)	8.0 (17.8%)	7.0 (14.3%)	4.0 (10.0%)	2.0 (11.8%)	27.0 (12.4%)
Western	13.0 (19.4%)	7.0 (15.6%)	5.0 (10.2%)	5.0 (12.5%)	3.0 (17.6%)	33.0 (15.1%)
Southern	3.0 (4.5%)	4.0 (8.9%)	3.0 (6.1%)	0.0 (0.0%)	2.0 (11.8%)	12.0 (5.5%)
Eastern	1.0 (1.5%)	2.0 (4.4%)	3.0 (6.1%)	2.0 (5.0%)	1.0 (5.9%)	9.0 (4.1%)
Northern	44.0 (65.7%)	24.0 (53.3%)	31.0 (63.3%)	29.0 (72.5%)	9.0 (52.9%)	137.0 (62.8%)

**Table 5:** Univariate and multivariate regression analyses of factors associated with stress scores.

Factor	Univariate			Multivariate		
	β	95% CI	*p*	β	95% CI	*p*
Maternal age, marital status, employment, nationality, education (all NS)						
No significant univariate associations—none entered in multivariate model						
Number of children (ref: one child)						
Two	2.46	[−2.03, 6.95]	0.282	-		
Three	1.50	[−2.95, 5.95]	0.507	-		
More than three	1.03	[−4.72, 6.78]	0.724	-		
More than one	−3.26	[−6.68, 0.16]	0.062	0.579	[−1.23, 2.39]	0.529
Family income (ref: <5000 SAR)						
5000–10,000 SAR	−5.82	[−9.32, −2.32]	0.001 *	0.279	[−1.59, 2.15]	0.769
>10,000 SAR	−2.91	[−7.94, 2.12]	0.256	-		
Residency region (ref: central—all NS)						
Western, Southern, Eastern, Northern—all *p* > 0.05				-		
Psychological scales						
Stress total score	0.702	[0.46, 0.95]	<0.001 *	0.113	[−0.03, 0.26]	0.124
Anxiety total score	0.730	[0.66, 0.81]	<0.001 *	0.317	[0.22, 0.41]	<0.001 *
Depression total score	0.785	[0.72, 0.85]	<0.001 *	0.522	[0.43, 0.62]	<0.001 *

* Statistically significant at *p* < 0.05; β = unstandardised coefficient; NS = not significant; CI = confidence interval.

**Table 6:** Categorisation of anxiety score levels.

Variable	Normal	Mild	Moderate	Severe	Ext. Severe	Total
Maternal age						
<20 years	0.0%	11.1%	0.0%	5.3%	3.3%	2.3%
20–29 years	20.4%	33.3%	19.6%	26.3%	35.6%	27.5%
30–39 years	44.4%	44.4%	41.3%	47.4%	35.6%	40.4%
>40 years	35.2%	11.1%	39.1%	21.1%	25.6%	29.8%
Marital status						
Married	90.7%	100%	84.8%	94.7%	90.0%	89.9%
Divorced/separated	9.3%	0.0%	15.2%	5.3%	8.9%	9.6%
Widow	0.0%	0.0%	0.0%	0.0%	1.1%	0.5%
Occupational status						
Employed	40.7%	22.2%	52.2%	47.4%	40.0%	42.7%
Unemployed	59.3%	77.8%	47.8%	52.6%	60.0%	57.3%
Nationality						
Saudi	94.4%	88.9%	97.8%	94.7%	87.8%	92.2%
Non-Saudi	5.6%	11.1%	2.2%	5.3%	12.2%	7.8%
Educational level						
<High school	16.7%	33.3%	13.0%	10.5%	11.1%	13.8%
High school	31.5%	11.1%	30.4%	26.3%	24.4%	27.1%
Bachelor’s degree	48.1%	55.6%	50.0%	47.4%	57.8%	52.8%
Postgraduate	3.7%	0.0%	6.5%	15.8%	6.7%	6.4%
Number of children						
One	14.8%	11.1%	19.6%	15.8%	7.8%	12.8%
Two	18.5%	33.3%	6.5%	36.8%	33.3%	24.3%
Three	20.4%	11.1%	23.9%	21.1%	32.2%	25.7%
>Three	46.3%	44.4%	50.0%	26.3%	26.7%	37.2%
Number of children with ASD						
One	98.1%	88.9%	95.7%	94.7%	92.2%	94.5%
More than one	1.9%	11.1%	4.3%	5.3%	7.8%	5.5%
Family income						
<5000 SAR	18.5%	0.0%	13.0%	15.8%	30.0%	21.1%
5000–10,000 SAR	27.8%	66.7%	47.8%	57.9%	42.2%	42.2%
>10,000 SAR	53.7%	33.3%	39.1%	26.3%	27.8%	36.7%
Residency region						
Central	5.6%	11.1%	19.6%	26.3%	10.0%	12.4%
Western	18.5%	33.3%	15.2%	5.3%	13.3%	15.1%
Southern	3.7%	0.0%	2.2%	5.3%	8.9%	5.5%
Eastern	1.9%	11.1%	4.3%	10.5%	3.3%	4.1%
Northern	70.4%	44.4%	58.7%	52.6%	64.4%	62.8%

**Table 7:** Univariate and multivariate regression analyses of factors associated with anxiety scores.

Factor	Univariate			Multivariate		
	β	95% CI	*p*	β	95% CI	*p*
Maternal age, marital status, employment, nationality, education (all NS)						
No significant univariate associations—none entered in multivariate model						
Number of children (ref: one child)						
Two	6.11	[1.36, 10.9]	0.012 *	4.30	[1.46, 7.14]	0.003 *
Three	3.82	[−0.89, 8.53]	0.111	3.17	[0.33, 6.00]	0.029 *
More than three	−0.77	[−5.23, 3.69]	0.735	1.71	[−1.02, 4.44]	0.218
Family income (ref: <5000 SAR)						
5000–10,000 SAR	−3.63	[−7.32, 0.06]	0.053	−0.04	[−2.38, 2.29]	0.970
>10,000 SAR	−7.17	[−11.0, −3.40]	<0.001 *	−1.21	[−3.63, 1.22]	0.327
Residency region (all NS—not entered in multivariate)						
Western, Southern, Eastern, Northern—all *p* > 0.05						
Psychological scales						
Stress total score	0.729	[0.46, 1.00]	<0.001 *	0.070	[−0.11, 0.25]	0.450
Anxiety total score	0.861	[0.77, 0.95]	<0.001 *	0.520	[0.36, 0.68]	<0.001 *
Depression total score	0.777	[0.69, 0.86]	<0.001 *	0.332	[0.18, 0.48]	<0.001 *

* Statistically significant at *p* < 0.05; β = unstandardised coefficient; NS = not significant; CI = confidence interval.

**Table 8:** Demographic characteristics of mothers of children with ASD according to depression severity level (N = 218).

Variable	Normal	Mild	Moderate	Severe	Ext. Severe	Total
Maternal age—dominant group						
30–39 years	44.8%	28.6%	49.1%	32.4%	33.3%	40.4%
Marital status—dominant group						
Married	94.0%	76.2%	91.2%	88.2%	89.7%	89.9%
Employment						
Unemployed	59.7%	47.6%	59.6%	50.0%	61.5%	57.3%
Nationality						
Saudi	92.5%	100%	98.2%	82.4%	87.2%	92.2%
Education—dominant group						
Bachelor’s degree	50.7%	47.6%	54.4%	52.9%	56.4%	52.8%
Number of children—dominant group						
>3 children	46.3%	52.4%	42.1%	29.4%	12.8%	37.2%
Income—dominant group						
5000–10,000 SAR	35.8%	57.1%	52.6%	35.3%	35.9%	42.2%
<5000 SAR (Ext. Severe)	14.9%	9.5%	19.3%	23.5%	38.5%	21.1%
Residency—dominant group						
Northern region	67.2%	57.1%	63.2%	52.9%	66.7%	62.8%

**Table 9:** Univariate and multivariate regression analyses of factors associated with depression scores.

Factor	Univariate			Multivariate		
	β	95% CI	*p*	β	95% CI	*p*
Maternal age (ref: <20 yrs)						
20–29 years	−3.47	[−13.1, 6.16]	0.479	-		
30–39 years	−6.05	[−15.6, 3.45]	0.211	-		
>40 years	−6.15	[−15.8, 3.45]	0.208	-		
Marital status (ref: married)						
Divorced/separated	1.00	[−3.77, 5.78]	0.679	-		
Widow	7.77	[−13.1, 28.6]	0.464	-		
Occupational status (ref: unemployed)						
Employed	−0.45	[−3.30, 2.39]	0.754	-		
Nationality (ref: non-Saudi)						
Saudi	−4.58	[−9.79, 0.63]	0.085	-		
Education (ref: <high school)						
High school	0.76	[−3.87, 5.39]	0.747	−1.12	[−3.56, 1.33]	0.369
Bachelor’s degree	1.64	[−2.60, 5.87]	0.447	−0.34	[−2.62, 1.95]	0.771
Postgraduate degree	6.71	[0.02, 13.4]	0.049 *	2.04	[−1.52, 5.59]	0.260
Number of children (ref: one child)						
Two	0.70	[−4.02, 5.42]	0.770	−2.06	[−4.63, 0.51]	0.116
Three	−0.64	[−5.32, 4.03]	0.787	−2.05	[−4.58, 0.48]	0.112
More than three	−5.37	[−9.80, −0.94]	0.018 *	−3.97	[−6.33, −1.61]	0.001 *
Number of children with ASD (ref: one)						
More than one	3.14	[−3.02, 9.29]	0.316	-		
Family income (ref: <5000 SAR)						
5000–10,000 SAR	−4.26	[−7.93, −0.60]	0.023 *	−0.63	[−2.69, 1.44]	0.549
>10,000 SAR	−6.42	[−10.2, −2.67]	<0.001 *	−0.22	[−2.42, 1.99]	0.846
Residency region (ref: central)						
Western	−1.54	[−6.93, 3.85]	0.573	-		
Southern	3.69	[−3.52, 10.9]	0.314	-		
Eastern	2.74	[−5.25, 10.7]	0.500	-		
Northern	−0.84	[−5.22, 3.53]	0.704	-		
Psychological scales						
Stress total score	0.707	[0.44, 0.97]	<0.001 *	0.031	[−0.13, 0.19]	0.703
Anxiety total score	0.903	[0.83, 0.98]	<0.001 *	0.671	[0.55, 0.79]	<0.001 *
Depression total score	0.758	[0.67, 0.84]	<0.001 *	0.246	[0.13, 0.36]	<0.001 *

* Statistically significant at *p* < 0.05.

## Data Availability

The data presented in this study are available on request from the corresponding author. The data are not publicly available due to privacy and ethical restrictions.

## Abbreviations

The following abbreviations are used in this manuscript:

ASD	Autism Spectrum Disorder
AFEQ	Autism Family Experience Questionnaire
DASS-21	Depression, Anxiety, and Stress Scale-21 Items
SAR	Saudi Riyals
